# Difference in determinants of ICU admission and death among COVID-19 hospitalized patients in two epidemic waves in Portugal: possible impact of healthcare burden and hospital bed occupancy on clinical management and outcomes, March–December 2020

**DOI:** 10.3389/fpubh.2023.1215833

**Published:** 2023-06-29

**Authors:** Vasco Ricoca Peixoto, André Vieira, Pedro Aguiar, Carlos Carvalho, Daniel Thomas, Paulo Sousa, Carla Nunes, Alexandre Abrantes

**Affiliations:** ^1^NOVA National School of Public Health, Public Health Research Centre, Comprehensive Health Research Center, CHRC, NOVA University Lisbon, Lisbon, Portugal; ^2^Unit for Multidisciplinary Research in Biomedicine (UMIB), School of Medicine and Biomedical Sciences (ICBAS), University of Porto, Porto, Portugal; ^3^Communicable Disease Surveillance Centre, Public Health Wales, Cardiff, United Kingdom

**Keywords:** COVID-19, health outcomes, risk factors, death, intensive care unit, hospital bed occupancy rate, healthcare burden, patient to physician ratio

## Abstract

**Aim:**

Identify factors associated with COVID-19 intensive care unit (ICU) admission and death among hospitalized cases in Portugal, and variations from the first to the second wave in Portugal, March–December 2020.

**Introduction:**

Determinants of ICU admission and death for COVID-19 need further understanding and may change over time. We used hospital discharge data (ICD-10 diagnosis-related groups) to identify factors associated with COVID-19 outcomes in two epidemic periods with different hospital burdens to inform policy and practice.

**Methods:**

We conducted a retrospective cohort study including all hospitalized cases of laboratory-confirmed COVID-19 in the Portuguese NHS hospitals, discharged from March to December 2020. We calculated sex, age, comorbidities, attack rates by period, and calculated adjusted relative risks (aRR) for the outcomes of admission to ICU and death, using Poisson regressions. We tested effect modification between two distinct pandemic periods (March–September/October–December) with lower and higher hospital burden, in other determinants.

**Results:**

Of 18,105 COVID-19 hospitalized cases, 10.22% were admitted to the ICU and 20.28% died in hospital before discharge. Being aged 60–69 years (when compared with those aged 0–49) was the strongest independent risk factor for ICU admission (aRR 1.91, 95%CI 1.62–2.26). Unlike ICU admission, risk of death increased continuously with age and in the presence of specific comorbidities. Overall, the probability of ICU admission was reduced in the second period but the risk of death did not change. Risk factors for ICU admission and death differed by epidemic period. Testing interactions, in the period with high hospital burden, those aged 80–89, women, and those with specific comorbidities had a significantly lower aRR for ICU admission. Risk of death increased in the second period for those with dementia and diabetes.

**Discussion and conclusions:**

The probability of ICU admission was reduced in the second period. Different patient profiles were identified for ICU and deaths among COVID-19-hospitalized patients in different pandemic periods with lower and higher hospital burden, possibly implying changes in clinical practice, priority setting, or clinical presentation that should be further investigated and discussed considering impacts of higher burden on services in health outcomes, to inform preparedness, healthcare workforce planning, and pandemic prevention measures.

## Introduction

Early studies of clinical results of COVID-19 in patients in China (1), Italy (2, 3), and the United States of America (4) have described risk factors for poorer clinical outcomes, including age, sex, and comorbidities. Identifying these determinants while adjusting for confounding factors can help inform clinical risk stratification and implementation of public health measures and improve epidemiological scenarios and forecasts on needed healthcare resources.

Several studies have focused on risk factors for ICU admission and death among hospitalized patients with COVID-19 (5).

Although hospitalized patients are only a fraction of the total cases in the general population, they provide quality data on comorbidities and outcomes and may give important information on clinical course and clinical management in different epidemic periods.

Advanced age, male sex, obesity, immunosuppression, and diabetes have been previously identified as risk factors for ICU admission (6, 7). Asthma and COPD were not identified as risk factors for ICU admission and death related to SARS-CoV2 infection (8).

Independent factors associated with in-hospital mortality included older age groups, generally above 50, being male, immunosuppression, renal disease, chronic lung disease, cardiovascular disease, neurologic disorders, diabetes, and dementia (9, 10). Identifying these features among patients in routine clinical practice may improve COVID-19 management (11). However, various studies on risk factors for severe outcomes of COVID-19 included small series of patients, mostly in single hospital centers (12–14).

One of the largest initial studies in hospital patients showed higher risk of death for patients with increased age, cardiac, pulmonary, and kidney disease, as well as malignancy, dementia, and obesity (15). The OpenSAFELY Collaborative study (16), in the United Kingdom, which included 17 million adult COVID-19 patients, added the findings that living in a more socio-economically deprived community was a relevant risk factors for death by COVID-19 and reinforced that older age, male sex, and having various other prior medical conditions were also relevant. High-quality data on possible risk factors for poorer outcomes among hospitalized COVID-19 patients is needed to inform clinical management, public health policy, and preparedness.

However, there is little research (5) on how risk factors may change over time in different pandemic periods considering healthcare burden. This may have implications related to clinical management and preventive intervention priorities, resource allocation, and healthcare supply. Various studies have shown increased risks of more severe outcomes in periods with higher hospital burden, higher number of patients per healthcare worker, or higher hospital occupancy rate (17–20).

We aim to further understand COVID-19 risk factors for two outcomes, namely ICU admission and death in hospitalized cases in Portugal, while comparing two epidemic periods with different healthcare burden, bed occupancy, and healthcare worker to patient ratio to generate hypotheses on its causes to inform future research, policy, and practice regarding peak pandemic and hospital burden periods, health workforce planning, and pandemic and seasonal preventive measures.

## Methods

### Study design and data sources

A retrospective cohort study including all hospitalized COVID-19 cases in Portuguese National Health Services (NHS) hospitals from March to December 2020 was conducted to identify factors associated with ICU admission and death in two different periods with different healthcare burdens.

### Data sources

All hospitalization diagnoses in NHS Hospitals are coded by trained medical doctors from clinical registries using the ICD-10 coding system. These ICD-10 codes are sequenced according to ICD-10 guidelines, considering specific COVID-19 guidelines for ICD-10 coding (21). Variables such as sex, age in years, and outcomes (which include death and transfer to ICU) are recorded as well as dates of admission and of outcome. A database with all admissions with COVID-19 U07.1 as a diagnosis was shared with the National School of Public Health by the Health Ministry services under a protocol for anonymized data sharing with academia during the COVID-19 pandemic.

### Case definition

A confirmed case of COVID-19 is anyone with a positive RT-PCR result for SARS-CoV-2 RNA in nasopharyngeal and/or oropharyngeal specimens regardless of clinical or epidemiological criteria. Included cases had a U07.1-COVID-19 diagnosis. This code does not include asymptomatic COVID-19, in the context of screening or acquired in the hospital.

### Outcomes

We considered two primary outcomes for all patients admitted to hospital with a COVID-19 diagnosis: admission to ICU and death during hospitalization.

### Exposures

**Figure 1:**
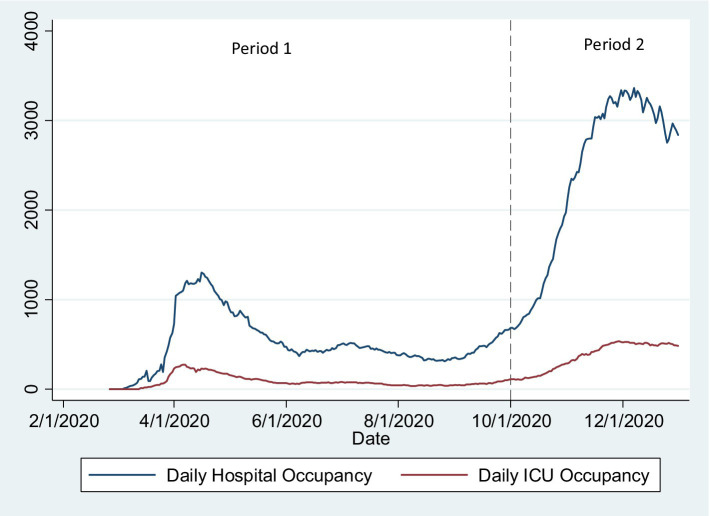
Number of hospital (general ward) and ICU occupied beds from 2 March to 31 December. Period 1 and 2 are visually represented. March–December 2020, Portugal.

We included in the analysis sex (Female/Male), age (recoded into “under 50,” “over 89,” and 10-year bands between 50 and 89 years of age), and comorbidities as independent variables/exposures. Dementia was categorized using the Charlston (22) comorbidity index. Mental/behavioral disease and pregnancy were categorized considering the corresponding ICD-10 category. Other ICD-10 codes were categorized considering Elixhauser (23) comorbidity index categories (24) if there were more than 100 observations in the category. We used these comorbidities index categories to avoid arbitrary inclusion of ICD-10 codes in categories of relevant comorbidities. We present adjusted models for those categories. We defined two Periods: since the first reported case in Portugal (2 March) to September 30; and from 1 October to 16 December. These correspond, respectively, to the first wave and summer period (low incidence and hospitalizations) and the second COVID-19 wave in Portugal (high incidence of cases, hospitalizations, and ICU admission and high hospital and ICU bed occupancy rate; Figure 1).

### Statistical analysis

We calculated attack rates (proportion of each outcome by stratum) by period. We calculated adjusted relative risk for ICU admission and death, by age group, sex, relevant comorbidities, and period, and tested interaction between the Period and other variables using robust Poisson regressions. To produce the final model with interaction terms between the period and the other variables we also tested for statistical significance of interaction terms. After that we created a model that included all interaction terms that were statistically significant when added individually to the fully adjusted model. Finally, we conducted backward elimination of non-significant interaction terms.

Statistical analysis was conducted in Stata (version 14, StataCorp, College Station, Texas, United States). All analyses used 95% CI and considered a *p-*value < 0.05 as statistically significant.

### Ethical considerations

Anonymized data were shared with the National School of Public Health-NOVA University of Lisbon by services of the Ministry of Health under a protocol for COVID-19 research.

## Results

Of 18,105 hospitalized cases of COVID-19, 1.852 were admitted to the ICU (10.22%) and 3.671 died in hospital before discharge (20.28%).

Attack rates for ICU admission where higher for those aged 60–69 and progressively lower after that age. Attack rates for death increase continuously with age. Proportion of hospitalized patients who were admitted to the ICU was higher in the first period in most patient characteristics (Table 1).

**Table 1:** Attack rates (proportion by stratum) for the outcome ICU and death in period 1, and period 2, Portugal, March–December 2020 (*n* = 18,105).

	(unite cells)						Death					
	Period 1 (March–September2020)			Period 2 (October–December 2020)			Period 1 (March–September2020)			Period 2 (October–December 2020)		
Exposure	Total	ICU	%	Total	ICU	%	Total	Death	%	Total	Death	%
**Sex**												
Female	4,440	387	**8.72**	4,153	208	**5.01**	4,440	815	**18.36**	4,153	887	**21.36**
Male	4,736	737	**15.56**	4,776	520	**10.89**	4,736	942	**19.89**	4,776	1,027	**21.50**
**Age**												
0–49	1933	180	**9.31**	1,255	96	**7.65**	1933	35	**1.81**	1,255	20	**1.59**
50–59	1,058	183	**17.30**	1,040	127	**12.21**	1,058	64	**6.05**	1,040	57	**5.48**
60–69	1,457	300	**20.59**	1,572	233	**14.82**	1,457	173	**11.87**	1,572	176	**11.20**
70–79	1722	298	**17.31**	1983	195	**9.83**	1722	393	**22.82**	1983	430	**21.68**
80–89	2,136	148	**6.93**	2,278	75	**3.29**	2,136	710	**33.24**	2,278	845	**37.09**
≥90	870	15	**1.72**	801	2	**0.25**	870	382	**43.91**	801	386	**48.19**
**Comorbidities**												
Malignant neoplasm	795	88	**11.07**	609	33	**5.42**	795	288	**36.23**	609	237	**38.92**
Metastatic cancer	91	5	**5.49**	55	0	**0.00**	91	44	**48.35**	55	29	**52.73**
Mental illness	2,138	356	**16.65**	1977	186	**9.41**	2,138	419	**19.60**	1977	393	**19.88**
Pregnancy	367	7	**1.91**	265	5	**1.89**	367	1	**0.27**	265	3	**1.13**
Cardiac disease (heart failure)	1,607	174	**10.83**	1,515	101	**6.67**	1,607	548	**34.10**	1,515	534	**35.25**
Chronic kidney disease (renal failure)	1,521	162	**10.65**	1,326	61	**4.60**	1,521	446	**29.32**	1,326	447	**33.71**
Chronic pulmonary disease	666	112	**16.82**	768	59	**7.68**	666	173	**25.98**	768	201	**26.17**
Asthma	309	45	**14.56**	339	41	**12.09**	309	36	**11.65**	339	34	**10.03**
Chronic liver disease	661	156	**23.60**	624	78	**12.50**	661	136	**20.57**	624	149	**23.88**
Obesity	1811	362	**19.99**	2,130	247	**11.60**	1811	312	**17.23**	2,130	414	**19.44**
Dementia	1,387	40	**2.88**	1,203	12	**1.00**	1,387	496	**35.76**	1,203	524	**43.56**
Hypertension	2,588	300	**11.59**	2,649	220	**8.31**	2,588	678	**26.20**	2,649	754	**28.46**
Diabetes	2069	279	**13.48**	2,191	178	**8.12**	2069	471	**22.76**	2,191	573	**26.15**
Neurologic disease	561	77	**13.73**	509	23	**4.52**	561	175	**31.19**	509	177	**34.77**
Total	9,176	1,124	**12.25**	8,929	728	**8.15**	9,176	1757	**19.15**	8,929	1914	**21.44**

### Determinants of ICU admission

**Figure 2:**
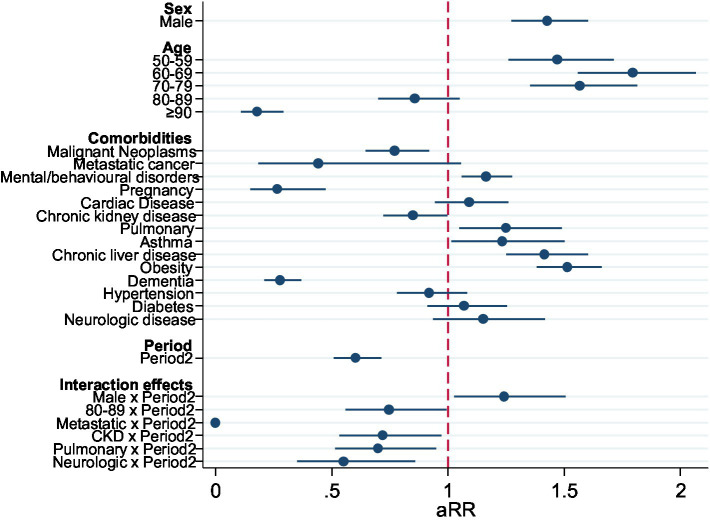
Forest plot representing the aRR of the adjusted model for the outcome ICU admission, including significant interactions between Period and other variables, Portugal, March–December 2020 (*n* = 18,105).

In multivariable analysis, there was an increase in probability of overall admission to ICU in age groups 50–59 and 60–69, and then decreasing above that age. The comorbidities with higher adjusted RR for admission to ICU were Obesity, Chronic Liver Disease, Mental Disease, and Asthma. Malignant neoplasms, Chronic Kidney Disease (CKD), and dementia reduced the probability of ICU admission. The risk of ICU admission was significantly lower in Period 2 (Table 2). When testing interactions with the Period, in the second period there was a significant increase in the adjusted risk of ICU admission for men, and a reduction for those aged 80–89 and those with metastatic cancer, CKD, pulmonary disease, and neurologic disease (Figure 2).

**Table 2:** Fully adjusted model for the outcome of the ICU admission including significant interactions between the Period and other variables, Portugal, March–December 2020 (*n* = 18,105).

**ICU admission**	**aRR**	***p*-value**	**[95%conf.interval]**
**Sex**			
Female			Ref
Male	1.43	<0.001	[1.27–1.60]
**Age**			
0–49			Ref
50–59	1.47	<0.001	[1.26–1.72]
60–60	1.80	<0.001	[1.56–2.07]
70–79	1.57	<0.001	[1.35–1.82]
80–89	0.86	0.138	[0.70–1.05]
≥90	0.18	<0.001	[0.11–0.29]
**Comorbidities**			
Malignant neoplasms	0.77	0.004	[0.65–0.92]
Metastatic cancer	0.44	0.067	[0.18–1.06]
Mental/behavioral disease	1.16	0.001	[1.06–1.28]
Pregnancy	0.27	<0.001	[0.15–0.47]
Cardiac disease (heart failure)	1.09	0.233	[0.95–1.26]
Chronic kidney disease (renal failure)	0.85	0.047	[0.72–1.00]
Chronic pulmonary disease	1.25	0.012	[1.05–1.49]
Asthma	1.23	0.035	[1.02–1.50]
Chronic liver disease	1.42	<0.001	[1.25–1.60]
Obesity	1.52	<0.001	[1.38–1.66]
Dementia	0.28	<0.001	[0.21–0.37]
Hypertension	0.92	0.317	[0.78–1.08]
Diabetes	1.07	0.408	[0.91–1.25]
Neurologic disorders	1.15	0.181	[0.94–1.42]
**Period**			
Period2	0.60	<0.001	[0.51–0.71]
**Effect modification by the period**			
Male*Period2	1.24	0.026	[1.03–1.51]
80–89*Period2	0.75	0.047	[0.56–1.00]
Metastatic*Period2	0.00	<0.001	[0.00–0.00]
CKD (Renal failure)*Period2	0.72	0.032	[0.53–0.97]
Pulmonary*Period2	0.70	0.022	[0.52–0.95]
Neurologic*Period2	0.55	0.009	[0.35–0.86]

### Risk factors for death

We observed that there was large, continuous increase of risk of death with age, unlike what was seen with ICU admissions.

In multivariable analysis, there was a continuous increase of risk of death with increasing age. Comorbidities that increase the risk of death were Malignant Neoplasms, Metastatic cancer, Cardiac Disease, CKD, Chronic Liver Disease, Dementia, and Neurologic Disease. Pregnancy, Asthma, and Diabetes reduced the risk of death, although diabetes had only a mild reduction. Adjusted risk of death did not change significantly in the second period although death rate was higher in the second period.

**Figure 3:**
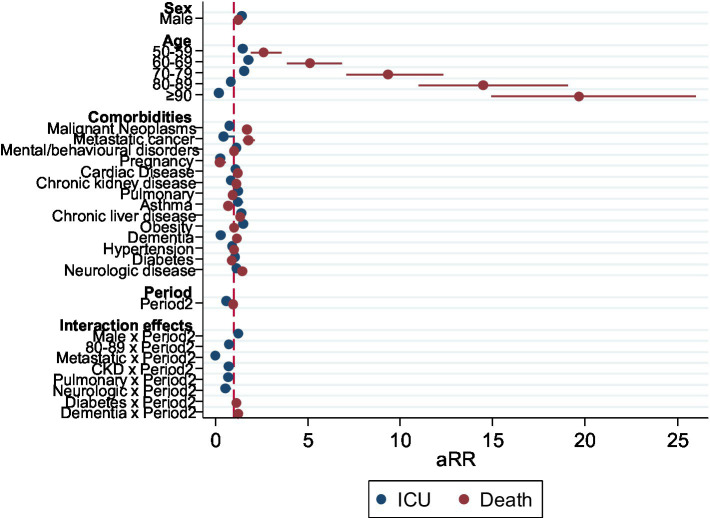
Forest plot representing the aRR of the adjusted model for the outcome death, including significant interactions between Period and other variables, Portugal, March–December 2020 (*n* = 18,105).

When testing interaction terms between the period and other variables for the outcome death, only the interaction of the second period with diabetes and with Dementia were statistically significant. They remained significant in a fully adjusted model with all previously significant interaction terms (Figure 3; Table 3).

**Table 3:** Fully adjusted model for the outcome death including interactions between Period and other variables, Portugal, March–December 2020 (*n* = 18,105).

**Death**	**aRR**	***p-*value**	**[95% conf. interval]**
**Sex**			
Female			Ref
Male	1.24	<0.001	[1.18–1.32]
**Age**			
0–49			Ref
50–59	2.62	<0.001	[1.90–3.59]
60–60	5.15	<0.001	[3.86–6.86]
70–79	9.35	<0.001	[7.09–12.34]
80–89	14.49	<0.001	[11.00–19.08]
≥90	19.69	<0.001	[14.91–25.99]
**Comorbidities**			
Malignant neoplasms	1.71	<0.001	[1.57–1.85]
Metastatic cancer	1.78	<0.001	[1.47–2.15]
Mental/behavioral disease	1.02	0.58	[0.95–1.09]
Pregnancy	0.23	<0.001	[0.09–0.58]
Cardiac disease (heart failure)	1.24	<0.001	[1.15–1.34]
Chronic kidney disease (renal failure)	1.16	<0.001	[1.09–1.24]
Chronic pulmonary disease	0.94	0.18	[0.86–1.03]
Asthma	0.69	<0.001	[0.56–0.85]
Chronic liver disease	1.36	<0.001	[1.23–1.51]
Obesity	1.03	0.42	[0.96–1.10]
Dementia	1.17	<0.001	[1.07–1.28]
Hypertension	1.02	0.58	[0.94–1.11]
Diabetes	0.89	0.03	[0.81–0,99]
Neurologic disease	1.46	<0.001	[1.34–1.60]
**Period**			
Period2	0.98	0.59	[0.91–1.05]
**Effect modification by the period**			
Diabetes*Period2	1.16	0.02	[1.03–1.30]
Dementia*Period2	1.26	0.00	[1.12–1.41]

## Discussion

We identified different adjusted risk factors for ICU admission and death in two different periods with different hospital burden, considering age, sex, and comorbidities. Obesity and Respiratory disease were significant risk factors for ICU admission but not for death. Risk of ICU increased with age but, unlike for death, risk of ICU admission stopped increasing after 70–79 years of age. Risk of ICU and death for age, sex, and various comorbidities changed in the second period of higher hospital burden. Testing effect modification by the period, in the period with high hospital burden those aged 80–89, women, and those with specific comorbidities had a significantly lower aRR for ICU admission. Risk of death increased in the second period for those with dementia and diabetes.

These findings identify knowledge gaps regarding ICU admission and clinical practice in periods of higher hospital burden. Changes may be due to the impact of healthcare burden in clinical management and ICU admission threshold, and variation in severity profile of patients admitted to hospital with COVID-19.

By observing variation in risk for different exposures in different periods we hypothesize that there may be changes in clinical care or severity profile of hospitalized patients in different time periods that can affect the probability of being admitted to an ICU and alter risk of death in some patients. In the face of strained resources and high bed occupancy rate, ICU admission may deprioritize patients with lower expected benefit from ICU admission. This may justify why patients aged 80–89 were less likely to be admitted to the ICU in the second period as well as those with relevant comorbidities. Patients over 90 and those with dementia did not see a difference in ICU admission probability in the two periods, possibly because they already had low admission rates due to low expected benefit, or because they die without ICU admission criteria or by causes and in clinical contexts that may not constitute ICU admission criteria. Similarly, increased risk of death for patients with dementia and diabetes may be due to changes in management related to hospital overload, implying that these patients may be of higher vulnerability when admitted to hospital in context of high hospital burden. A higher baseline severity or clinical vulnerability of patients admitted to hospital in the second period due to various factors may also contribute to the observed increase in risk of death for specific patient profiles.

Age remains the most relevant risk factor after adjustment. In our study, some comorbidities were weak risk factors or not associated with increased risk of death, such as chronic pulmonary disease, obesity, and diabetes. This may be partly because categorization using Elixhauser comorbidity index may include less severe disease or eventually because some patients in those categories may be admitted with less severe profile in a precautionary approach. In the adjusted model, obesity and respiratory disease were not significant risk factors for death but were strong risk factors for ICU admissions among comorbidities. These findings may imply lower thresholds for admission to the ICU for these patients but not intrinsically higher severity, although this must be considered with caution since obesity and respiratory disease have been risk factors for death in other studies (25, 26). In our study it is possible that Elixhauser chronic pulmonary disease and diabetes categories includes less severe disease. Specifically, diabetes includes complicated and uncomplicated subcategories. In the first period it is possible that respiratory disease had a lower admission threshold because of a precautionary approach since in the second period there was a large reduction in adjusted risk for ICU admission in those with respiratory disease. However, other comorbidities such as cardiac disease and chronic kidney disease did not increase risk of ICU admission but increased the risk of death, which raises further research questions related to patient ICU admission practices and outcomes.

We found that Neurologic Disease, Cancer, CKD, cardiac disease, liver disease, and dementia were important risk factors for death. Interestingly, the effect of dementia was modified by the period of analysis and the risk of death was even higher in the second period. This may be due to lower threshold for hospital admission of cases with dementia due to increase in hospital occupancy and due to changes in clinical management of cases outside of the hospital and of admitted cases that may have increased risk of death. It is also possible that disease severity in this group increased during the second period among cases in specific settings such as nursing homes in outbreak contexts; this could be related to variant severity profile, higher infectious dose exposure, or other non-observed factors related to pre-hospital and hospital clinical management.

Conversely, Cardiac Disease, CKD, malignant neoplasms, metastatic disease, and dementia had no association or a negative association with ICU admission but all increased the risk of death.

The risk of ICU admission decreases in older ages, as found in other Portuguese cohort studies of all confirmed cases in the first COVID-19 wave in Portugal (27). That reduction was larger during the second period (October–December). It is possible that some older patients may die without meeting criteria for ICU admission or eventually, many who end up meeting those criteria may die before they can be admitted. Furthermore, they may be no expected clinical benefit and very low recovery expectations if admitted to the ICU. Debate has been ongoing on this topic considering the challenges and ethics of admitting patients of a very advanced age to ICU, patient and family wishes, and therapeutic futility (28–32). Portuguese guidelines considered the existence of comorbidities for hospital admission but the criteria for admission to the ICU include mainly clinical severity criteria (33) which could impact the estimates for comorbidities for the two outcomes.

Combined, these findings suggest that there are differences in ICU admission thresholds for different patient characteristics or different expected benefits from admission, in different periods with higher and lower hospital and bed occupancy rates. This should remain relevant even if severity profile at admission changed for specific patient profiles. Few studies describe outcomes of ICU admission for different patient profiles with COVID-19 and continuous research in this area remains necessary.

This study has strengths and limitations. We used information extracted from the national electronic records of patients’ hospitalizations that uses ICD-10 codes for comorbidities (coded by trained medical doctors after discharge, from clinical notes, through a standardized procedure in accordance with ICD-10 coding guidelines) (21). This assures good quality of comorbidity and outcome data.

By using Elixhauser (34) comorbidity Index categories, we intend to reduce arbitrariness in categorizing ICD-10 comorbidities by relying on a validated comorbidity index used to predict risk of severe outcomes. This may also facilitate replication of studies. A recent study compared Charlston Comorbidity Index categories and Elixhauser categories and found similar comorbidity risks for death (35). However, as previously described, using Elixhauser categories may include a range of clinical entities and clinical severities in some groups, that may include milder comorbidities, possibly underestimating risk for more specific comorbidities within those categories.

There are limitations in this study. Firstly, hospitalized patient characteristics at admission may have changed over time. This means that risk estimates for outcomes are subject to variation by this factor as previously discussed. For example, the first hospitalized patients in Portugal were hospitalized without severe disease for isolation purposes. However, this situation comprises a minimal number of cases in the first week of cases in Portugal and cannot produce relevant selection bias. However, in different periods, the clinical thresholds for general hospital admission may have varied differentially for different patient characteristics due to higher hospital burden and stretch of capacity. This could contribute to a more severe clinical profile at admission for specific comorbidities and age groups that could influence the change in risk estimates for ICU admission and death in the two analyzed periods.

Used data comprised all patients with a COVID-19 diagnosis registered in ICD-10 (21). It is possible that some patients were admitted for other conditions not related to COVID-19 infection. However, during this period of the pandemic, most hospitalized patients with COVID-19 were admitted due to COVID-19 symptoms, decompensation of chronic disease due to COVID-19 infection, or complications arising from the infection. Still, in theory, this could introduce bias in estimates of risk if COVID-19-infected patients with other reasons for admission had a systematically lower or higher risk of ICU admission or death. If patients with specific ages or comorbidities had more often been admitted for a condition that was not related to COVID-19, and systematically had lower risk of ICU admission or death, this would underestimate risk for those ages or comorbidities. However, in our study, COVID-19 (U07.1) was sequenced as the first diagnosis in 15,299 admissions, the second in 1,075, and the third in 744. These comprise approximately 95% of analyzed admissions, making the analysis robust for the purpose of exploring differences in risk factors in two periods for the outcomes of interest for COVID-19 patients.

ICD-10 coding guidelines only reccomends code (U07.1) COVID-19 as first diagnosis when COVID-19 meets the definition of principal diagnosis, when the reason for the encounter/admission is a respiratory manifestation of COVID-19. COVID-19 that is identified in screening or acquired in hospital, asymptomatic COVID-19, and probable COVID-19 are not assigned the (U07.1) COVID-19 code. A patient with sepsis, obstetrics, or transplant complications due to COVID-19 will be coded with those codes as first diagnosis. Decompensation of a relevant comorbidity atributed to COVID-19 will usually imply COVID-19 as one of the first diagnosis.

There are relevant comorbidities that we could not adjust for that have been previously found to be of relevance for the COVID-19 severity outcomes, such as economic deprivation (16) and minority ethnic groups (16, 36).

This is one of few studies comparing how risk changed for ICU admission and death among hospitalized COVID-19 patients in periods with high and low hospital burden. Further research is warranted to understand possible changes in clinical practice for older patients, women, and patients with specific comorbidities in periods of higher hospital burden to inform human resource planning and health practice and policy. Chances of survival in ICU should be further researched to help priority setting in increased burden contexts while aiming to increase capacity to guarantee access to all who may benefit from ICU and hospital admission in general. Further research is needed to understand how increase in hospital burden and bed occupancy may impact admissions and probability of ICU admission or death and what type of patients may have larger benefit from ICU admission. Personal and family considerations, as well as clinical judgment, are necessary to make decisions on a case-by-case approach but epidemiological sound data should contribute to these decisions.

Evidence that increased hospital burden has a negative impact on clinical management and outcomes for both COVID and non-COVID-conditions is relevant to inform control measures facing scenarios of increased hospital burden by COVID and other infectious respiratory diseases or due to reduced human resources. When facing increased burden, healthcare professionals will inevitably have to manage limited technical and human resources.

## Conclusion

In this study, age was the strongest single risk factor for death among hospitalized patients. For ICU admission, after 60–69 years, the risk of ICU admissions starts reducing and becomes protective after 80 years old, but risk of death increases continuously with age. In the second analyzed period probability of ICU admission was significantly lower, specially for older age groups, women, and those with specific comorbidities and the risk of death increased in the second period, only for those with dementia and diabetes. These findings may imply changes only, in clinical practice due to increased hospital burden or changes in clinical severity of hospitalized patients with specific characteristics, and should be further investigated. Further research on changes in determinants of admission, ICU admission, and death may improve understanding on how different severity profiles, increased hospital burden, or reduction of healthcare workforce and increased patient-staff ratio may affect clinical practice and outcomes to inform preparedness, healthcare workforce planning, prevention measures, and healthcare practice and policy.

## Data availability statement

The data analyzed in this study is subject to the following licenses/restrictions: anonymized national hospital admissions database, shared with academia under a COVID-19 research protocol. Requests to access these datasets should be directed to geral@acss.min-saude.pt.

## Author contributions

VR, AV, PA, AA, and CN: study design. VR and PA: analysis. VR: writing of first draft. VR, AV, PA, CC, DT, PS, CN, and AA: reviews and other significant contributions. All authors contributed to the article and approved the submitted version.

## Funding

This work was funded by Fundação Ciência e Tecnologia, IP national support through CHRC (UIDP/04923/2020).

## In memoriam

In Memoriam of Professor Carla Nunes.

## Conflict of interest

The authors declare that the research was conducted in the absence of any commercial or financial relationships that could be construed as a potential conflict of interest.

## Publisher’s note

All claims expressed in this article are solely those of the authors and do not necessarily represent those of their affiliated organizations, or those of the publisher, the editors and the reviewers. Any product that may be evaluated in this article, or claim that may be made by its manufacturer, is not guaranteed or endorsed by the publisher.

## References

[1] Guan W Ni Z Hu Y Liang WH Ou CQ He JX. Clinical characteristics of coronavirus disease 2019 in China. N Engl J Med. (2020) 382:1708–20. doi: 10.1056/NEJMoa2002032, PMID: 32109013 PMC7092819

[2] Onder G Rezza G Brusaferro S. Case-fatality rate and characteristics of patients dying in relation to COVID-19 in Italy. JAMA. (2020) 323:1775–6. doi: 10.1001/jama.2020.4683, PMID: 32203977

[3] Livingston E Bucher K. Coronavirus disease 2019 (COVID-19) in Italy. JAMA. (2020) 323:1335–5. doi: 10.1001/jama.2020.4344 32181795

[4] Bialek S Boundy E Bowen V. Severe outcomes among patients with coronavirus disease 2019 (COVID-19)—United States, February 12-march 16, 2020. Morb Mortal Wkly Rep. (2020) 69:343–6. doi: 10.15585/mmwr.mm6912e2, PMID: 32214079 PMC7725513

[5] Ferland L Carvalho C Gomes Dias J Lamb F Adlhoch C Suetens C. Risk of hospitalization and death for healthcare workers with COVID-19 in nine European countries, January 2020–January 2021. J Hosp Infect. (2022) 119:170–4. doi: 10.1016/j.jhin.2021.10.015, PMID: 34752802 PMC8665668

[6] van Halem K Bruyndonckx R van der Hilst J Cox J Driesen P Opsomer M. Risk factors for mortality in hospitalized patients with COVID-19 at the start of the pandemic in Belgium: a retrospective cohort study. BMC Infect Dis. (2020) 20:897. doi: 10.1186/s12879-020-05605-3, PMID: 33246431 PMC7691970

[7] Ayaz A Arshad A Malik H Ali H Hussain E Jamil B. Risk factors for intensive care unit admission and mortality in hospitalized COVID-19 patients. Acute Crit Care. (2020) 35:249–54. doi: 10.4266/ACC.2020.00381, PMID: 33172229 PMC7808857

[8] Calmes D Graff S Maes N Frix AN Thys M Bonhomme O. Asthma and COPD are not risk factors for ICU stay and death in case of SARS-CoV2 infection. J Allergy Clin Immunol Pract. (2021) 9:160–9. doi: 10.1016/j.jaip.2020.09.044, PMID: 33038592 PMC7539890

[9] Kim L Garg S O’Halloran A Whitaker M Pham H Anderson EJ. Risk factors for intensive care unit admission and in-hospital mortality among hospitalized adults identified through the US coronavirus disease 2019 (COVID-19)-associated hospitalization surveillance network (COVID-NET). Clin Infect Dis. (2020) 72:e206–14. doi: 10.1093/cid/ciaa1012, PMID: 32674114 PMC7454425

[10] Filardo TD Khan MR Krawczyk N Galitzer H Karmen-Tuohy S Coffee M. Comorbidity and clinical factors associated with COVID-19 critical illness and mortality at a large public hospital in new York City in the early phase of the pandemic (march-April 2020). PLoS One. (2020) 15:e0242760. doi: 10.1371/journal.pone.0242760 33227019 PMC7682848

[11] Kaeuffer C Le Hyaric C Fabacher T Mootien J Dervieux B Ruch Y. Clinical characteristics and risk factors associated with severe COVID-19: prospective analysis of 1,045 hospitalised cases in north-eastern France, march 2020. Eur Secur. (2020) 25:2000895. doi: 10.2807/1560-7917.ES.2020.25.48.2000895, PMID: 33272355 PMC7716399

[12] Grasselli G Zangrillo A Zanella A Antonelli M Cabrini L Castelli A. Baseline characteristics and outcomes of 1591 patients infected with SARS-CoV-2 admitted to ICUs of the Lombardy region, Italy. JAMA. (2020) 323:1574–81. doi: 10.1001/jama.2020.5394, PMID: 32250385 PMC7136855

[13] Korean Society of Infectious Diseases and Korea Centers for Disease Control and Prevention. Analysis on 54 mortality cases of coronavirus disease 2019 in the Republic of Korea from January 19 to march 10, 2020. J Korean Med Sci. (2020) 35:e132. doi: 10.3346/JKMS.2020.35.E132, PMID: 32233161 PMC7105509

[14] Zhou F Yu T Du R Fan G Liu Y Liu Z. Clinical course and risk factors for mortality of adult inpatients with COVID-19 in Wuhan, China: a retrospective cohort study. Lancet. (2020) 395:1054–62. doi: 10.1016/S0140-6736(20)30566-3, PMID: 32171076 PMC7270627

[15] Docherty AB Harrison EM Green CA Hardwick H Pius R Norman L. Features of 16,749 hospitalised UK patients with COVID-19 using the ISARIC WHO clinical characterisation protocol. medRxiv [Preprint]. 10.1136/bmj.m1985 PMC7243036 32444460

[16] Collaborative TO Williamson E Walker AJ Bhaskaran K Bacon S Bates C Morton C. E.. OpenSAFELY: factors associated with COVID-19-related hospital death in the linked electronic health records of 17 million adult NHS patients. (2020). medRxiv [Preprint].

[17] Presanis AM Kunzmann K Grosso FM Jackson CH Corbella A Grasselli G. Risk factors associated with severe hospital burden of COVID-19 disease in Regione Lombardia: a cohort study. BMC Infect Dis. (2021) 21:1–16. doi: 10.1186/S12879-021-06750-Z/FIGURES/13 34620121 PMC8496148

[18] Grimm C Dickel S Sachkova A Popp M Golinksi M Fichtner F. Targeted minimal staff-to-patient ratios are unachievable – a Nationwide survey in German ICUs during the COVID-19 pandemic. Cureus. (2021) 13:e15755. doi: 10.7759/CUREUS.15755, PMID: 34290932 PMC8289403

[19] Harvey PR Trudgill NJ. The association between physician staff numbers and mortality in English hospitals. EClinicalMedicine. (2021) 32:100709. doi: 10.1016/J.ECLINM.2020.100709, PMID: 33681734 PMC7910697

[20] Aiken LH Sloane DM Bruyneel L van den Heede K Griffiths P Busse R. Nurse staffing and education and hospital mortality in nine European countries: a retrospective observational study. Lancet. (2014) 383:1824–30. doi: 10.1016/S0140-6736(13)62631-8, PMID: 24581683 PMC4035380

[21] CDC. Comprehensive listing ICD-10-CM files. https://www.cdc.gov/nchs/icd/Comprehensive-Listing-of-ICD-10-CM-Files.htm (Accessed 29 May 2023).

[22] Deyo RA Cherkin DC Ciol MA. Adapting a clinical comorbidity index for use with ICD-9-CM administrative databases. J Clin Epidemiol. (1992) 45:613–9. doi: 10.1016/0895-4356(92)90133-8, PMID: 1607900

[23] Cai M Liu E Zhang R Lin X Rigdon SE Qian Z. Comparing the performance of charlson and elixhauser comorbidity indices to predict in-hospital mortality among a Chinese population. Clin Epidemiol. (2020) 12:307–16. doi: 10.2147/CLEP.S241610, PMID: 32256119 PMC7090198

[24] Quan H Sundararajan V Halfon P Fong A Burnand B Luthi JC. Coding algorithms for defining comorbidities in ICD-9-CM and ICD-10 administrative data. Med Care. (2005) 43:1130–9. doi: 10.1097/01.MLR.0000182534.19832.83, PMID: 16224307

[25] Sawadogo W Tsegaye M Gizaw A Adera T. Overweight and obesity as risk factors for COVID-19-associated hospitalisations and death: systematic review and meta-analysis. BMJ Nutr Prev Heal. (2022) 5:10–8. doi: 10.1136/BMJNPH-2021-000375, PMID: 35814718 PMC8783972

[26] Aveyard P Gao M Lindson N Hartmann-Boyce J Watkinson P Young D. Association between pre-existing respiratory disease and its treatment, and severe COVID-19: a population cohort study. Lancet Respir Med. (2021) 9:909–23. doi: 10.1016/S2213-2600(21)00095-3, PMID: 33812494 PMC8016404

[27] Peixoto VR Vieira A Aguiar P Sousa P Carvalho C Thomas D. Determinants for hospitalisations, intensive care unit admission and death among 20,293 reported COVID-19 cases in Portugal, march to April 2020. Eur Secur. (2021) 26:2001059. doi: 10.2807/1560-7917.ES.2021.26.33.2001059/CITE/PLAINTEXT PMC8380973 34414882

[28] Nguyen Y-L Angus DC Boumendil A Guidet B. The challenge of admitting the very elderly to intensive care. Ann Intensive Care. (2011) 1:29. doi: 10.1186/2110-5820-1-29, PMID: 21906383 PMC3224497

[29] Monteiro F. Mechanical ventilation and medical futility or dysthanasia, the dialectic of high technology in intensive medicine. Rev Port Pneumol. (2006) 12:281–92. doi: 10.1016/S0873-2159(15)30431-1, PMID: 16967178

[30] Lima C. Medicina High Tech, Obstinação Terapêutica e Distanasia. Med Int. (2006) 13:79–82.

[31] Ranzani OT Besen BAMP Herridge MS. Focus on the frail and elderly: who should have a trial of ICU treatment? Intensive Care Med. (2020) 46:1030–2. doi: 10.1007/s00134-020-05963-1, PMID: 32123988

[32] Guidet B Leblanc G Simon T Woimant M Quenot JP Ganansia O. Effect of systematic intensive care unit triage on long-term mortality among critically ill elderly patients in France a randomized clinical trial. JAMA. (2017) 318:1450–9. doi: 10.1001/jama.2017.13889, PMID: 28973065 PMC5710364

[33] da Saúde Direção-Geral. Norma no 004/2020 de 23/03/2020 atualizada a 25/04/2020—COVID-19: FASE DE MITIGAÇÃO – Abordagem do Doente com Suspeita ou Infeção por SARS-CoV-2. Available at: https://www.dgs.pt/directrizes-da-dgs/normas-e-circulares-normativas/norma-n-0042020-de-23032020-pdf.aspx (Accessed 27 May 2020).

[34] Gulliford MC Prevost AT Charlton J Juszczyk D Soames J McDermott L. Effectiveness and safety of electronically delivered prescribing feedback and decision support on antibiotic use for respiratory illness in primary care: REDUCE cluster randomised trial. BMJ. (2019) 364:l236. doi: 10.1136/bmj.l236, PMID: 30755451 PMC6371944

[35] Rosenthal N Cao Z Gundrum J Sianis J Safo S. Risk factors associated with in-hospital mortality in a US National Sample of patients with COVID-19. JAMA Netw Open. (2020) 3:e2029058. doi: 10.1001/jamanetworkopen.2020.29058, PMID: 33301018 PMC7729428

[36] Khunti K Singh AK Pareek M Hanif W. Is ethnicity linked to incidence or outcomes of covid-19? BMJ. (2020):369. doi: 10.1136/bmj.m1548 32312785

